# Supplementary material for: Voltage-based magnetization switching and reading in magnetoelectric spin-orbit nanodevices
Source: Nat Commun. 2024 Mar 1;15:1902. doi: 10.1038/s41467-024-45868-x (PMC10907725; doi:10.1038/s41467-024-45868-x)
Supplement: Supplementary file 1 — Supplementary Information [file 41467_2024_45868_MOESM1_ESM.pdf]

Voltage-based magnetization switching and reading in magnetoelectric spin-orbit nanodevices

-SUPPLEMENTARY INFORMATION-

Diogo C. Vaz[1,♦], Chia-Ching Lin[2], John Plombon[2], Won Young Choi[1,*], Inge Groen[1], Isabel C. Arango[1], Andrey Chuvilin[1,3], Luis E. Hueso[1,3], Dmitri E. Nikonov[2], Hai Li[2], Punyashloka Debashis[2], Scott B. Clendenning[2], Tanay A. Gosavi[2], Yen-Lin Huang[4], Bhagwati Prasad[4,5], Ramamoorthy Ramesh[4], Aymeric Vecchiola[6], Manuel Bibes[6], Karim Bouzehouane[6], Stephane Fusil[6], Vincent Garcia[6], Ian A. Young[2], Fèlix Casanova[1,3,♦]

[1] CIC nanoGUNE BRTA, 20018, Donostia-San Sebastian, Basque Country, Spain

[2] Components Research, Intel Corp., Hillsboro, Oregon 97124, USA

[3] IKERBASQUE, Basque Foundation for Science, 48009 Bilbao, Basque Country, Spain

[4] Department of Physics, University of California, Berkeley, California 94720, USA

[5] Materials Engineering Department, Indian Institute of Science, Bengaluru, 560012, Karnataka, India

[6] Unité Mixte de Physique, CNRS, Thales, Université Paris-Saclay, 91767, Palaiseau, France

[*] Current affiliation: VanaM Inc., 21-1 Doshin-ro 4-gil, Yeongdeungpo-gu, Seoul, Republic of Korea

[♦] Correspondence to: d.vaz@nanogune.eu, f.casanova@nanogune.eu

Supplementary Note 1: Fabrication of MESO device.

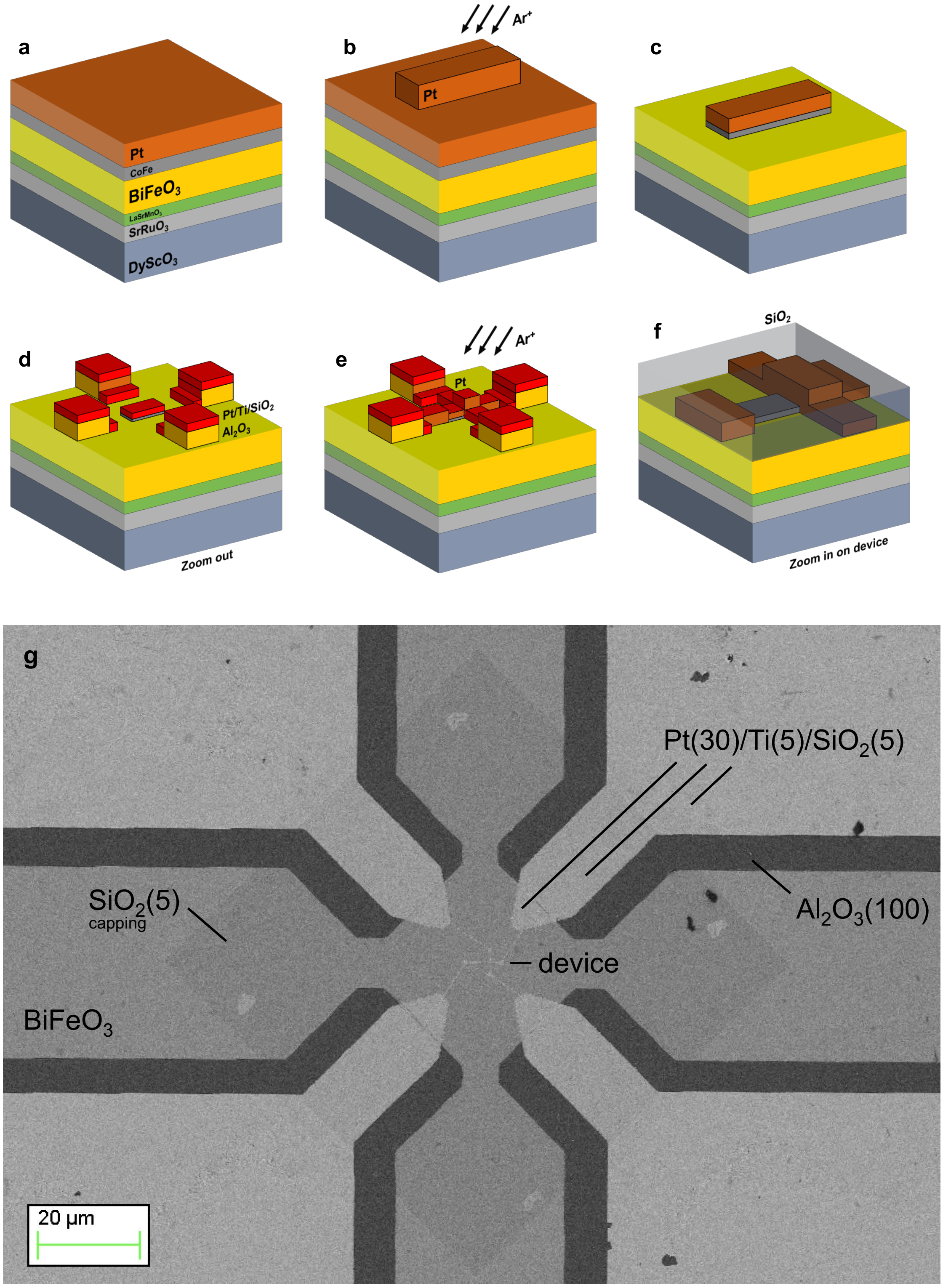

Figure S1 – Fabrication workflow of MESO nanodevices. a, Initial full stack. **b**, Patterning of sacrificial Pt nanowire and Ar-ion milling. **c**, Resulting Pt/CoFe wire. **d**, Patterning of contact pads with Al_2O_3 and Pt/Ti/SiO_2. **e**, Patterning Pt T-shape on the Pt/CoFe wire. **f**, Ar-ion milling of the whole sample and capping with SiO_2. **g**, SEM image of the final device.

We start the fabrication of the MESO nanodevices from a full stack of Pt(10 nm)/CoFe(2.5 nm)/$BiFeO_3$(30 nm)/$La_{0.7}Sr_{0.3}MnO_3$(4 nm)/$SrRuO_3$(10 nm)//$DyScO_3$ (Fig. S1a), deposited and grown with a combination of magnetron sputtering (for the deposition of Pt and CoFe) and pulsed laser deposition (for $BiFeO_3$, $La_{0.7}Sr_{0.3}MnO_3$, and $SrRuO_3$). The first step of fabrication is the positive e-beam lithography patterning of a nanowire of Pt using PMMA 950A2, onto the initial full stack, which will act as a "sacrificial wire" (Fig. S1b and S1c). The thickness of this wire is 15 nm. Then, Ar-ion milling is used to etch the whole sample, where the time it takes to remove the initial Pt/CoFe layer is the same as for the removal of the fabricated Pt wire. We have opted for this "sacrificial" layer procedure in order to avoid issues of negative resist removal, recurrently observed in lithography processes below 200nm. Then, two positive e-beam lithography steps using double-layer PMMA (495 A4 + 950 A2) are performed to pattern the buffer layer for the bonding pads, made of Al_2O_3(100 nm), and the contacting pads, made of Pt(40 nm)/Ti(5 nm)/SiO_2(5 nm), as seen in Fig. S1d. The use of Al_2O_3 was crucial in avoiding shortcuts between wire-bonded contacts and the $SrRuO_3$ bottom electrode. Pt/Ti is used to ensure good adhesion between the wire-bonded contact and the pad. SiO_2 is used to avoid electrical shortcuts in the areas where Pt/Ti and $BiFeO_3$ are in direct contact. A fourth positive e-beam lithography step is performed to define Pt(20 nm) nanowires, including the T-shaped nanostructure, which are aligned to the initial Pt/CoFe, creating a Pt-on-Pt junction that preserves the original in-situ interface between Pt and CoFe (Fig. S1e). Lastly, the whole sample is etched with Ar-ion milling, so that the Pt on top of the CoFe is removed. The samples are immediately capped with SiO_2(5 nm) to prevent CoFe oxidation (Fig. S1f). An SEM image of the full device (including contacts) is shown in Fig. S1g.

Supplementary Note 2: Extended PFM and MFM characterization.

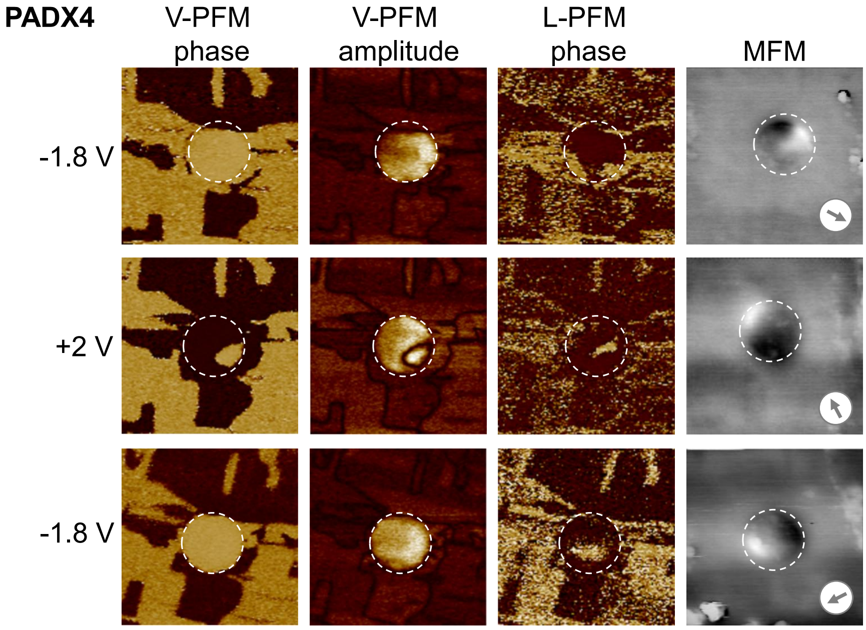

Figure S2 – Extended PFM and MFM data of Fig. 2 (main text). Out-of-plane (V) PFM phase and amplitude images and in-plane (L) PFM phase images after applying voltage pulses of −1.8 V, 2 V, and −1.8 V to a disk of Pt/CoFe with a diameter of 200 nm. Corresponding MFM images showing the magnetization direction of the CoFe after each pulse, represented by the grey arrows.

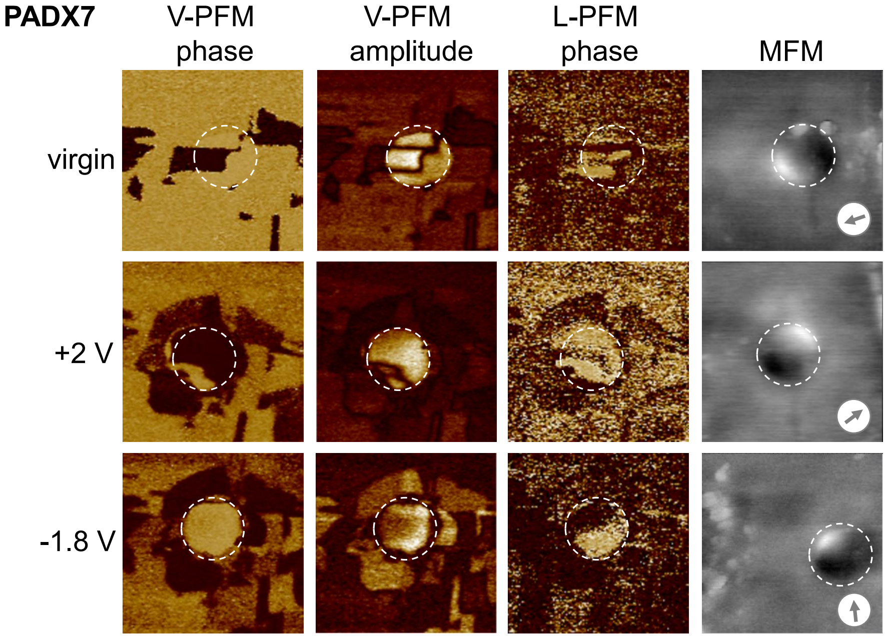

Figure S3 – Extended PFM and MFM characterization of device X7. Out-of-plane (V) PFM phase and amplitude images and in-plane (L) PFM phase images for a virgin state and after applying voltage pulses of 2 V and −1.8 V to a disk of Pt/CoFe with a diameter of 200 nm. Corresponding MFM images showing the magnetization direction of the CoFe after each pulse, represented by the grey arrows.

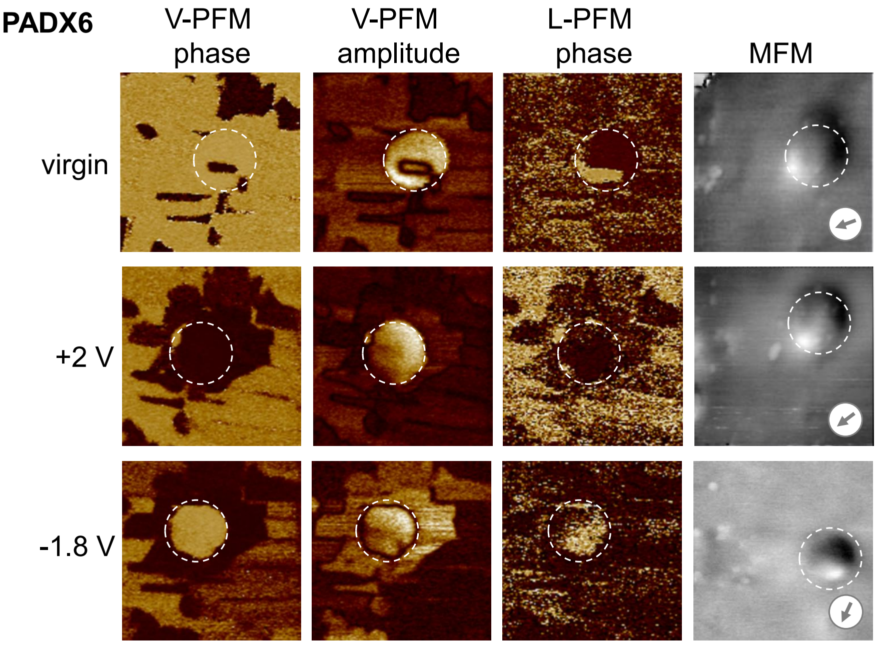

Figure S4 – Extended PFM and MFM characterization of device X6. Out-of-plane (V) PFM phase and amplitude images and in-plane (L) PFM phase images for a virgin state and after applying voltage pulses of 2 V and –1.8 V to a disk of Pt/CoFe with a diameter of 200 nm. Corresponding MFM images showing the magnetization direction of the CoFe after each pulse, represented by the grey arrows.

As stated in the main text, out of 9 devices probed (in the same sample) using PFM and MFM, we have observed that the magnetization could be switched only at times and for some devices. In Fig. S2, we show the extended data set for the device discussed in the main text, where magnetization reversal and partial rotation are observed after V_p=2 V and V_p=–1.8 V, respectively. Device X7, displayed in Fig. S3, showed similar behavior, although the same pulse polarity led to magnetization reversed from the bottom left orientation to the top right. For a subsequent V_p=–1.8 V pulse, the magnetization once again rotates, but does not go through a full reversal. We note here that the out-of-plane component of the polarization switches similarly between up and down, while the in-plane component remains rather unchanged, even though with a different contrast than the one shown in Fig. S2 (L-PFM). A third set of data is shown in Fig. S4, for device X6, as an example of a device where no magnetization reversal or rotation was observed, even though the polarization (both out-of-plane and in-plane) was observed to switch. These results emphasize the fact that the manipulation of the magnetization orientation cannot be only connected with the polarization state, and that full control of the magnetization direction will require additional information regarding the magnetic textures of the $BiFeO_3$ underneath the disks.

Supplementary Note 3: Initialization of the MESO devices

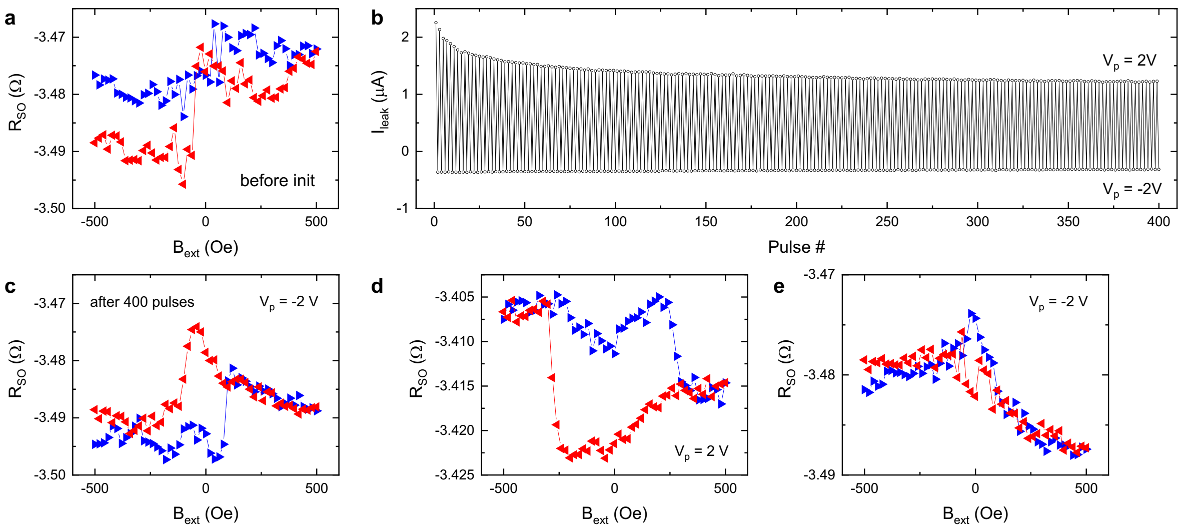

Figure S5 – Initialization of devices. a, R_{SO} vs. external magnetic field B_{ext} in the virgin device. **b**, Leakage current I_{leak} for alternating voltage pulses of $V_p=\pm2$ V. **c**, R_{SO} vs B_{ext} after initialization (last pulse $V_p=-2$ V). **d**, The following R_{SO} vs B_{ext} after $V_p=2$ V. **e**, The following R_{SO} vs B_{ext} after $V_p=-2$ V.

We have consistently observed that the magnetization loops and overall leakage of $BiFeO_3$ change after performing an initialization protocol, where consecutive voltage pulses with opposite polarity are applied. This is consistent with the well-known "wake up" phenomenon in ferroelectrics, related to the depinning of defect-pinned domain walls and removal of charged defects[1]. As shown in Fig. S5a, before initialization, the magnetization loop reveals that the CoFe wire may have pinned magnetic domain regions to the as-grown magnetic state of the $BiFeO_3$. After 400 pulses of $V_p=\pm2$ V, the leakage current I_{leak} for positive voltage pulses steadily decreases from 2 μA to around 1.25 μA (Fig. S5b). After this, the three possible states discussed in the main text can be observed (Fig. S5c, S5d, and S5e).

Supplementary Note 4: Correlation between electrical read-out and magnetization orientation.

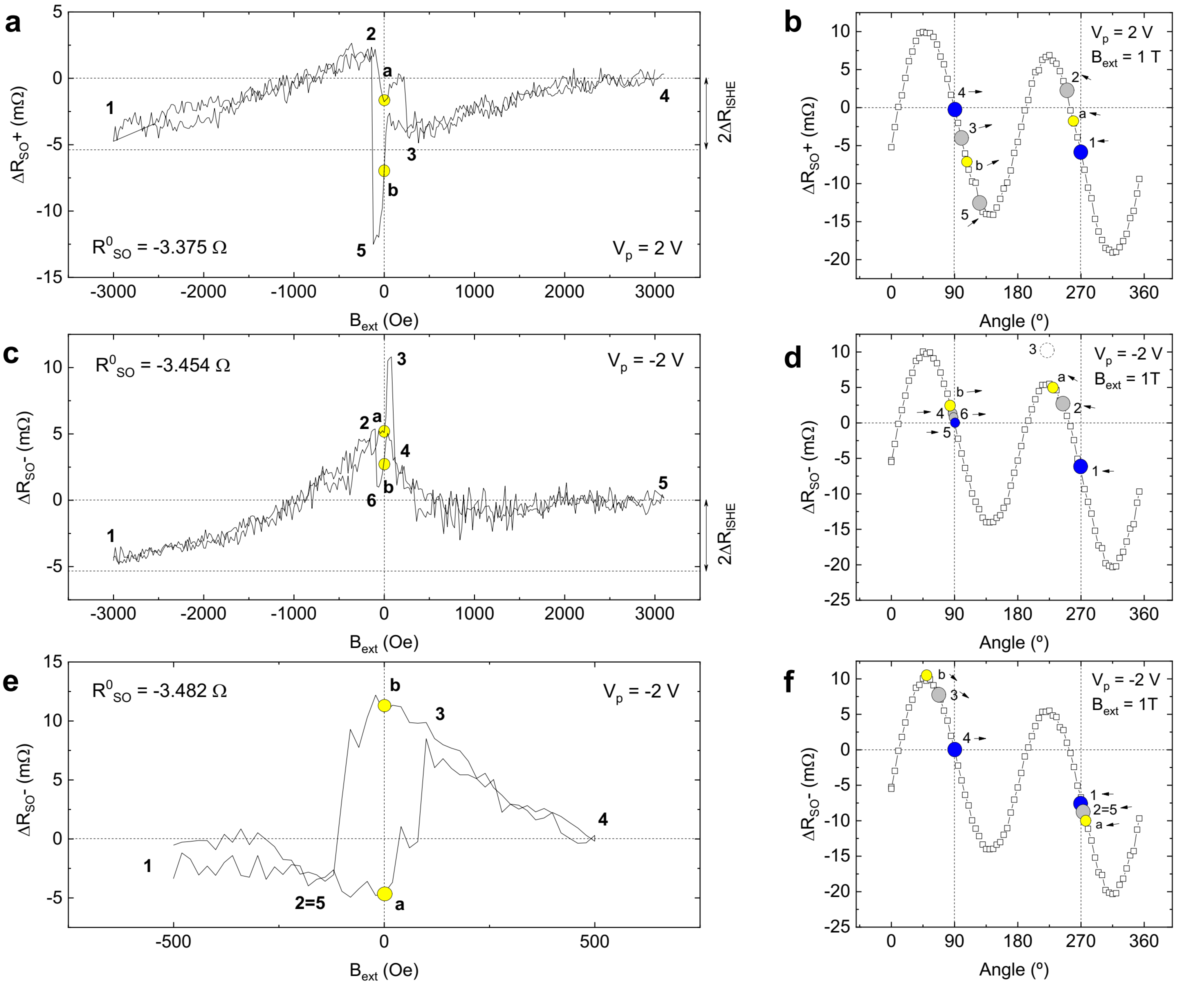

Figure S6 – Extended magnetization loops and corresponding R_{SO} angle dependence. $\Delta R_{SO}^{+(-)}=R_{SO}-R^0_{SO}$ vs. external magnetic field B_{ext} and corresponding ΔR_{SO} angle dependences with B_{ext}=1 T after **a, b**, V_p=2 V, **c, d,** V_p=–2 V (intermediate state), **e, f,** and V_p=–2 V, where R_{SO} is the raw output resistance, and R^0_{SO} the baseline resistance at large positive B_{ext}. The subscript + and – represent the ΔR_{SO} after V_p=2 V and V_p=–2 V, respectively. ΔR_{SO} values from each angle dependence are numbered and matched to the magnetization loops. Arrows represent the magnetization direction of the CoFe wire as seen from the top.

To obtain further information on which magnetization direction each resistance R_{SO} corresponds to, we have measured the angle dependence of ΔR_{SO} under a rotating in-plane magnetic field B_{ext}=1 T for V_p=±2 V. From ΔR_{SO} vs. B_{ext}, we can identify that ΔR_{SO} at 3000 Oe corresponds to the magnetization pointing to the right (90º, aligned with the CoFe wire long axis). From this point, we observe if ΔR_{SO} either increases or decreases when reducing the B_{ext} and match this increase/decrease with the angle dependence, which gives us information on the evolution of the magnetization direction.

Supplementary Note 5: Full data set for the magnetic switching after V_p=2 V.

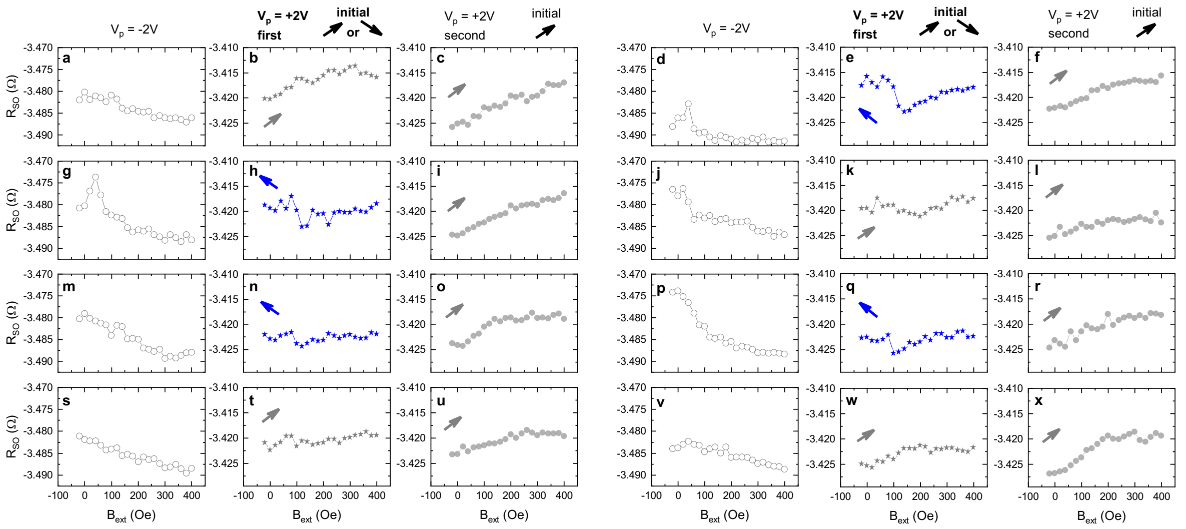

Figure S7 – Full electrical characterization of the magnetization switching for V_p=2 V. Panels **a** through **x** are shown in the experimental sequential order.

The magnetization is first initialized by applying V_p=-2 V (open circles), and the field is swept from 0 Oe to 400 Oe and back. This way, the orientation of the magnetization is known to be oriented towards the right. However, given the two possible states after V_p=-2 V, the initial magnetization orientation either points to the top right or bottom right (as shown by the bold arrows at the top). After a first V_p=2 V at B_{ext}=0 Oe, the magnetization is either observed to switch ~90º (blue stars) or not (grey stars), corresponding to the two R_{SO} branches of Fig. 3d. After collecting this curve, the field is swept back from 400 Oe to 0 Oe, so that the magnetization state now points to the top right. A subsequent V_p=2 V never drives additional magnetization rotation (grey full circles).

Supplementary Note 6: Full data set for the magnetic switching after V_p=–2 V.

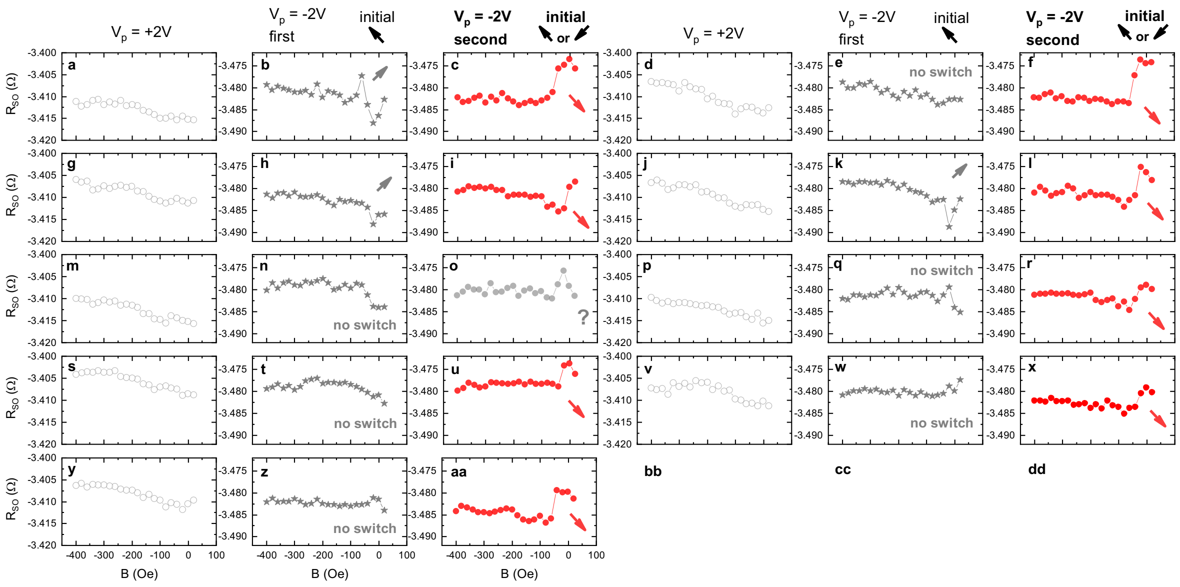

Figure S8 – Full electrical characterization of the magnetization switching for V_p=–2 V. Panels **a** through **aa** are shown in the experimental sequential order.

The magnetization is first initialized by applying V_p=2 V (open circles), and the field is swept from 0 Oe to –400 Oe and back. This way, the orientation of the magnetization is oriented towards the top left. Then, after a first V_p=–2 V at B_{ext}=0 Oe (grey stars), the magnetization is at times observed to switch by ~90° (Fig. S8b, S8h, S8k), given that the sweep to –400 Oe reveals a small jump in R_{SO} as seen in Fig. 3e. The remaining curves (labeled "no switch") may correspond to a slight rotation of the magnetization to the bottom left, as seen in Fig. 3f. After collecting this curve, the field is swept back from –400 Oe to 0 Oe, so that the magnetization state now points to either the top left or bottom left (as shown by the bold arrows at the top), depending on whether V_p=–2 V led to the intermediate of fully switched state (Fig. 3e or 3f, respectively). A subsequent V_p=–2 V now drives a magnetization reversal towards the right (red full circles), corresponding to the upper branch in Fig. 3f. This behavior is always observed, except for one instance (Fig. S8o) where it is unclear if the magnetization is fully reversed.

Supplementary Note 7: Full data set of the read-out resistance after V_p=±2, with and without a static magnetic field applied.

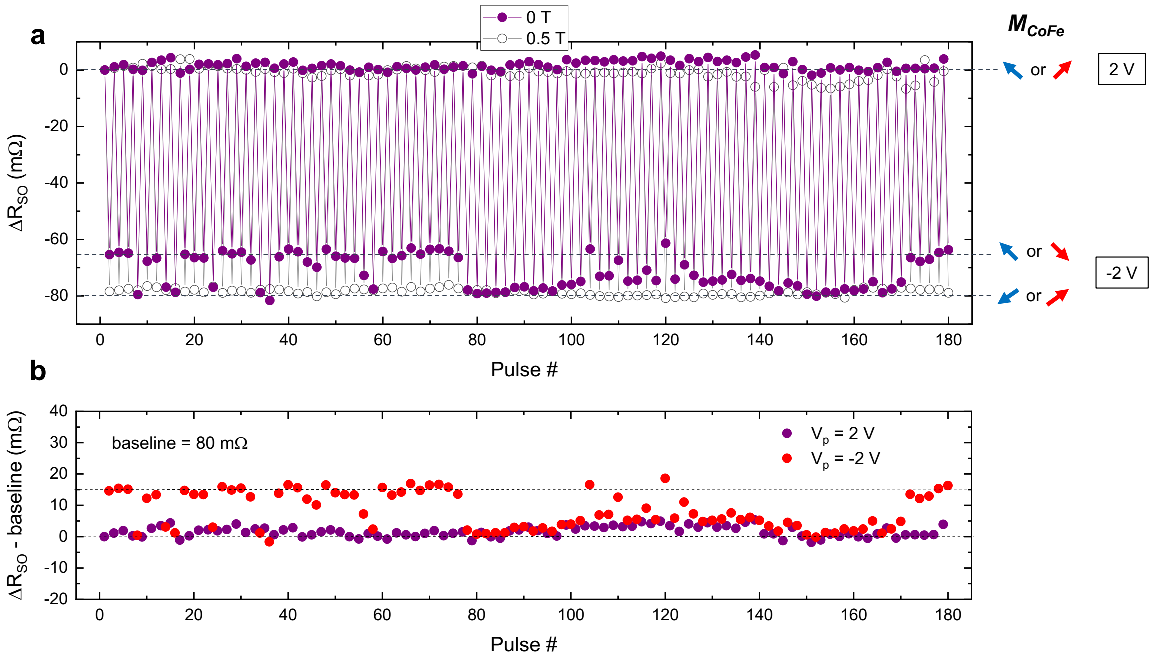

Figure S9 – Steady-state switching of the MESO device. a, ΔR_{SO} as a function of alternating V_p=±2 V at 0 T (purple filled circles) and 0.5 T (empty circles). Blue and red arrows on the right represent the possible CoFe magnetization orientations as seen from the top of the device. **b**, Same data as presented in panel a, but subtracted by the baseline contribution of about 80 mΩ.

Using the MESO device as an active element in circuits will require the observation of different output states for different magnetization direction. In Fig. S9a, we plot the output resistance ΔR_{SO}, normalized by the average output resistance after V_p=2 V. In the main text, although we have used a magnetic field sweep to verify the magnetization direction upon voltage pulse, switching and reading functionality of the MESO device should ideally be shown without any field applied, since that is how the device should work in a real circuit scenario.

We observe that, between voltage pulses of opposite polarity, ΔR_{SO} shifts around –80 mΩ, corresponding to the baseline shift discussed in the main text and shown in Fig. 3a. We reemphasize that the baseline shift is attributed to a slight modulation of the resistivity of CoFe and not magnetization switching.

However, on top of this shift, we observe that after V_p=–2 V two resistances values (ΔR_{SO}=–65 mΩ and ΔR_{SO}=–80 mΩ) are obtained. Without the external magnetic field sweep analysis, the two possible states observed in Fig. 4 (main text) make it difficult to identify to which magnetization direction each resistance value corresponds. As shown by the arrows next to the plot, ΔR_{SO}=–65 mΩ can represent magnetization pointing towards the top left or bottom

right, while ΔR_{SO}=–80 mΩ can represent magnetization pointing towards the bottom left or top right.

For V_p=2 V, where only one state is observed, the variability of ΔR_{SO} after each pulse is in the same order of magnitude as the expected R_{SO} difference between the two possible magnetization states (magnetization towards the top left or top right, as represented by the arrows).

Lastly, by performing the same experiment under an in-plane B_{ext}=0.5 T (open circles), i.e. fixing the magnetization direction in one direction throughout the whole experiment, ΔR_{SO}=–65 mΩ is never observed, suggesting that while the $BiFeO_3$ is still switching (leading to a baseline shift of ~80 mΩ), the extra change in ΔR_{SO} may be related with changes in the magnetization direction of the CoFe.

The change in ΔR_{SO} is further evidenced in Fig. S9b, where the baseline is subtracted to the data presented in panel a. Here, we observe that, at times, a shift of about 15 mΩ is observed upon application of a V_p=–2 V. However, given the uncertainty associated with different magnetization orientations corresponding to similar ΔR_{SO}, a magnetic field sweep is still required to ensure that magnetization switching occurred, as presented in Fig. 4 of the main text. As a counterexample, solely based on the ΔR_{SO} data presented here, we may have a tilt of the magnetization of <45º, which could represent a shift in ΔR_{SO} of about 15 mΩ (see Fig. 3c, in the main text).

Supplementary Note 8: Effect of voltage pulse duration on the switching of the $BiFeO_3$ and the switching of M_{CoFe}.

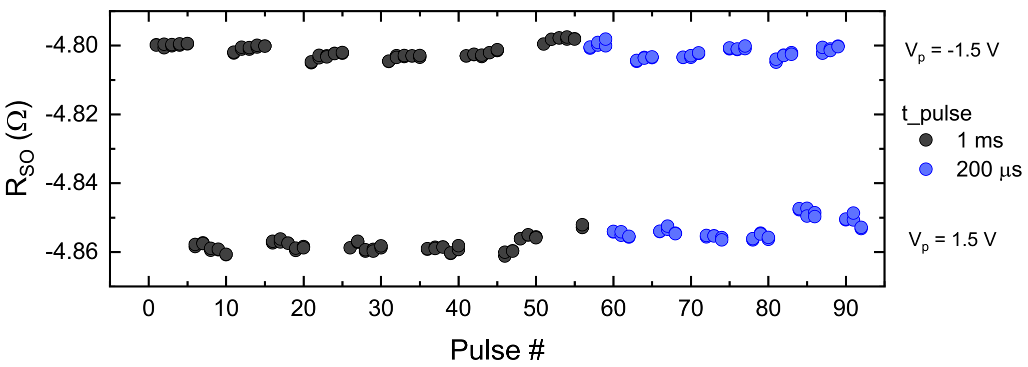

Figure S10 – Output resistance R_{SO} as a function of alternating V_p=±2 V at 0 T, for a pulse duration time of 1 ms (dark circles) and 200 μs.

All the switching experiments shown in the main text were performed with a voltage pulse duration of 200 μs, mostly due to the time constraints of running the long full set of experiments for several devices at different pulse durations. As displayed in Figure S10, we observed no change in the ability to switch the $BiFeO_3$ when increasing the voltage pulse duration from 200 μs to 1 ms. However, to minimize excessive current flow through the $BiFeO_3$, and to avoid the potential breakdown of this layer, we have used the minimum pulse duration experimentally possible by the equipment used.

However, the discussion on the effect of voltage pulse duration on these devices can be divided in two parts: 1) the switching dynamics of the $BiFeO_3$, and 2) the switching dynamics of the CoFe magnetic element upon switching the $BiFeO_3$.

1) It was experimentally demonstrated[2] that a 20-nm-thick $BiFeO_3$ can be switched with less than 10 ns voltage pulses, with pulse duration being linearly dependent on the lateral area of the top electrode. A rough comparison with our Pt/CoFe-based MESO devices reveals that with an approximate area of 190 μm^2 (accounting for the Pt nanowires that are also in direct contact with the $BiFeO_3$), our 30-nm-thick $BiFeO_3$ could, in principle, be switched with a pulse as short as ~50 ns.

2) Yet, it was theoretically shown that when considering a CoFe/$BiFeO_3$ system, the switching time to successfully switch the CoFe magnetic element also needs to be considered[3]. While the lowest theoretical switching time for $BiFeO_3$ was 30 ps, the minimum time required to switch the magnetic element was 1.45 ns, meaning that the multiferroic cannot switch too fast, or the M_{CoFe} will not be able to follow it. We note that these simulations were performed for a magnetic element of 40 nm x 20 nm (thickness of 2 nm), while our CoFe element is 500 nm x

150 nm (thickness of 2.5 nm), implying that the minimum switching time could be much larger in our case.

With this in mind, we expect that the 200 μs used in the experiments shown in the main text is well above the expected minimum switching time of M_{CoFe}, but not high enough to promote larger leakage or potential damage to the $BiFeO_3$ layer.

Supplementary Note 9: Coupling between different sketched CoFe magnetic elements with $BiFeO_3$.

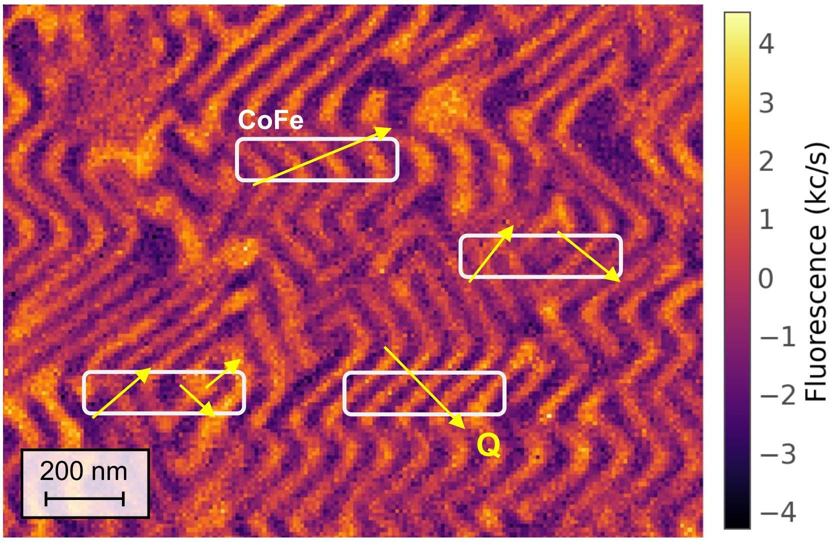

Figure S11 – Sketch of randomly distributed CoFe elements, to scale, with respect to the magnetic landscape of $BiFeO_3$.

The lack of device-to-device reproducibility can be attributed to the complex magnetic textures and spin cycloid probed by nitrogen-vacancy (NV) magnetometry and displayed in Fig. 5 of the main text. As sketched in Fig. S11, when fabricating the MESO devices, we have no knowledge of the position of the device with respect to this magnetic texture, leading to a situation where several devices with the exact same orientation, but fabricated a few microns apart, will experience completely different exchange coupling with $BiFeO_3$. To improve the reproducibility of these devices, the ferroelectric domain structure of $BiFeO_3$ needs to be engineered to ideally possess a single macroscopic domain region, with a coherent cycloid propagation that can be consistently switched. Alternatively, having no cycloid present, i.e., a uniform magnetic region where M_{CoFe} would couple with a uniform antiferromagnetic order in the multiferroic, could vastly simplify the reproducibility issues. Both approaches have been experimentally explored through strain and chemical doping[4], but have yet to be implemented with functional nanodevices.

References

1. Zhu, H. *et al.* Fatigue behavior of resistive switching in a $BiFeO_3$ thin film. *Jpn. J. Appl. Phys.* **57**, 041501 (2018).
2. Parsonnet, E. *et al.* Toward Intrinsic Ferroelectric Switching in Multiferroic $BiFeO_3$. *Phys. Rev. Lett.* **125**, 067601 (2020).

3. Liao, Y.-C. *et al.* Understanding the Switching Mechanisms of the Antiferromagnet/Ferromagnet Heterojunction. *Nano Lett.* **20**, 7919–7926 (2020).
4. Haykal, A. *et al.* Antiferromagnetic textures in $BiFeO_3$ controlled by strain and electric field. *Nat. Commun.* **11**, 1704 (2020).
